# The Impact of COVID-19 on Consumers’ Psychological Behavior Based on Data Mining for Online User Comments in the Catering Industry in China

**DOI:** 10.3390/ijerph18084178

**Published:** 2021-04-15

**Authors:** Chenyu Zhang, Jiayue Jiang, Hong Jin, Tinggui Chen

**Affiliations:** 1 School of Statistics and Mathematics, Zhejiang Gongshang University, Hangzhou 310018, China; zcybetty0407@163.com (C.Z.); jiangjiayue559@163.com (J.J.); izuzyz@163.com (H.J.); 2 Modern Business Research Center, Zhejiang Gongshang University, Hangzhou 310018, China

**Keywords:** consumers’ psychological behavior, data mining, online user comments, COVID-19

## Abstract

The outbreak of COVID-19 in late 2019 has had a huge impact on people’s daily life. Many restaurant businesses have been greatly affected by it. Consumers’ preferences for catering industry in China have changed, such as environmental hygiene, variety of dishes, and service methods. Therefore, the analysis of consumer preference differences and changes before and after the epidemic can not only provide emergency strategies for the catering industry but further improve the catering industry’s ability to deal with public health emergencies. This paper takes five cities in China as representatives to explore the impact of COVID-19 on China’s catering industry. Based on catering review data from August 2019 to April 2020, this paper first carries out Latent Dirichlet Allocation (LDA) topic analysis and SNOWNLP (A Python library for processing Chinese text) sentiment analysis. Then this paper compares the results of topic classification and sentiment analysis before and after the epidemic. Furthermore, differences and changes of consumer preferences are obtained and preferences of consumers under COVID-19 are analyzed and forecasted. The results of LDA thematic analysis before the outbreak of COVID-19 show that consumers tend to punch in cyber celebrity restaurants and pay more attention to the taste of dishes, whereas after it consumers pay more attention to the changes of dishes, dining environment as well as epidemic prevention. The number of packages and takeout was also increasing. However, the waiting time is constantly considered by consumers before and after COVID-19. Firstly, to our surprise, final outcome of emotional analysis showed that consumers’ emotional state was more positive after the epidemic than before. COVID-19 has changed the lifestyle of consumers, consumption concepts, and consumption habits. Therefore, businesses also need to take positive and flexible measures to actively get feedback from consumers to adjust dishes and business methods. Secondly, the psychological attitude of catering consumers is relatively positive during the epidemic period, which indicates that consumers have great confidence in the recovery and development of the catering industry. Businesses can comply with consumers’ psychology and combine consumption vouchers with restaurant discounts to promote consumers’ consumption. Finally, the environment and service play more and more important effect on consumers’ emotional scores at present, which indicates that dining state and comfortable mealtime environment are becoming increasingly valuable. Therefore, businesses need to improve service standards.

## 1. Background of Research

Small and medium-sized enterprises have encountered greater difficulties than ever before under the outbreak of COVID-19 at the end of 2019 [1]. Nowadays, people’s daily life has gradually recovered under the normal situation of epidemic prevention and control. However, the negative influences on economy have been seen continuing, especially for some small and medium-sized enterprises and individual businesses with weak pressure resistance [2]. At present, small and medium-sized enterprises as well as individual businesses in different forms can found everywhere in China. They are like the “capillaries” in China’s economic development. While meeting the daily needs of residents, they contribute more than 80% of employment, more than 70% of scientific and technological innovation achievements, more than 60% of GDP and more than 50% of tax revenue to the country [3]. Among these small and medium-sized enterprises, catering, accommodation, filmed entertainment, wholesale and retail, as well as transportation and tourism suffered the most concentrated and maximum effects [4]. They rely on crowd density and mobility to obtain income. However, sharply decrease of people flow under the epidemic makes their earnings almost zero. It is this situation that causes many enterprises drop into not only the problem of short-term capital liquidity but long-term financing depression.

According to statistics, the catering industry ranked second among the top ten industries that most affected by the epidemic [5]. The dinner on New Year’s Eve was almost entirely unsubscribed because of the outbreak of COVID-19. Affected by the epidemic, people also need to cut the time they go out when dining together is prohibited, which makes many restaurants suffer heavy losses [6]. In the first half of 2020, the national catering revenue was 1460.9 billion Yuan, a year-on-year decrease of 32.8%, and the growth rate was 42.2 percentage points lower than that of the same period last year. The catering revenue of units above designated size was 311.9 billion Yuan, a year-on-year decrease of 30.2% [7]. In 2020, the catering revenue was 16.6% lower than that of the previous year [8]. Numerous food produced go to hoarded, the pressure of rent, water, and electricity charges as well as labor cost increased rapidly, making the catering industry profits plummeted, facing a huge survival crisis [6].

## 2. Literature Review

The work resumptions of all walks of life have been widely concerned by the society. Since the outbreak of COVID-19, tertiary industry has been carried out investigation and research on the survival and development under the epidemic. Typical literatures are as follows: Yang and Han [9] conduct a “real-time” investigation with user-generated content on Twitter to identify the main concerns in the hospitality industry during the COVID-19 pandemic and gain a better understanding of the business challenges and responses from the industry. In terms of how the global pandemic applies to our eating patterns and eating habits, Michael [10] found that COVID-19 fundamentally changed many everyday food-related practices, whether due to supply chain failures or national directives (such as in-place shelter orders). At the same time, in the face of massive text data, many scholars use text mining technology, combined with machine language deep learning to form a more complete algorithm to study natural language processing [11]. For example, Tian et al. [12] used sensory analysis method to study the review data of the catering industry and found the internal relationship between customer emotion and customer rating. Kumar et al. [13] comprehensively surveyed the evolution of the online social networks, their associated risks, and solutions. The various security models and the state of the art algorithms had been discussed along with a comparative meta-analysis using machine learning, deep learning, and statistical testing to recommend a better solution. Lee et al. [14] proposed a novel unified approach for learning to rank products based on online product reviews. Unlike existing approaches, it used deep-learning techniques to extract the high-level latent review representation that contains the most semantic information in the learning process. The experiments showed that this approach outperformed the existing methods in sales rank prediction based on online product reviews. In addition, Wang et al. [15] proposed an unsupervised method for judging comment credibility based on the Biterm Sentiment Latent Dirichlet Allocation (BS-LDA) model. Their experimental results using comments from Amazon.com demonstrated that the overall performance of their approach could play an important role in determining the credibility of comments in some situation. However, there is still a lack of research on the change of consumers’ preference for the catering industry before and after COVID-19. As the main service object of the catering industry, consumers are an important force to promote the resumption of work and production, transformation, and upgrading of the catering industry.

According to this situation, this paper crawls review data in China from August 2019 to April 2020, and divides the data into “before the outbreak” and “after the outbreak” by taking January 2020 as the dividing point. Subsequently, through analyzing these comments by LDA topic analysis method, this paper compares the theme results and comes to the differences of consumer preferences before and after the outbreak. According to the score through the analysis of consumers’ emotion after the epidemic, this paper gets the consumers’ preference for catering after the epidemic, analyzing as well as predicting the consumers’ preference under public health emergencies, so as to provide emergency strategies for the catering industry and further improve the catering industry’s ability to deal with public health emergencies.

## 3. Proposed Methods and Its Implementation Process

In this paper, the authors use method of text mining to study the collected review data, apply LDA model (Latent Dirichlet Allocation) to construct the topic, and utilize SNOWNLP library to supplement the emotional analysis.

### 3.1. Theoretical Basis of LDA Model

LDA [16,17] is a document topic generation model, which is a three-layer Bayesian probability model including “word, topic, and document”. Its core idea is to represent the document abstractly as the probability distribution of the topic. Relationship between the document and the vocabulary are embodied in the implied topic.

Each word of LDA topic model is obtained through the process of selecting topics with a certain probability and selecting words from each topic with a certain probability. The core formula is as follows:

(1) $$p\left(\mathrm{words}|\mathrm{documents}\right)=\underset{topics}{\sum }p\left(\mathrm{words}|\mathrm{topics}\right)\times p(\mathrm{topics}|\mathrm{documents})$$

### 3.2. The Principle of Emotion Analysis Based on SNOWNLP

SNOWNLP is a Chinese natural language processing Python sentiment analysis library, with Chinese sentiment training set. It reads each line of text in the file through code, analyzes its sentiment and outputs the final result. It uses naive Bayesian principle [18,19] to comment and predict the data. This paper deals with the classification of comment data into two categories: positive (POS) and negative (NEG). Its features, $w_{1},w_{2},w_{3},\ldots ,w_{n}$, are independent of each other, and the formula is expressed as follows:

(2) $$p\left(pos|w_{1},\ldots ,w_{n}\right)=p\left(w_{1},\ldots ,w_{n}|pos\right)\times \frac{p\left(pos\right)}{p\left(w_{1},\ldots ,w_{n}\right)}$$

(3) $$p\left(neg|w_{1},\ldots ,w_{n}\right)=p\left(w_{1},\ldots ,w_{n}|neg\right)\times \frac{p\left(neg\right)}{p\left(w_{1},\ldots ,w_{n}\right)}$$

(4) $$p\left(w_{1},\ldots ,w_{n}\right)=p\left(w_{1},\ldots ,w_{n}|pos\right)\times p\left(pos\right)+p\left(w_{1},\ldots ,w_{n}|neg\right)\times p\left(neg\right)$$

### 3.3. Implement Process

Step 1: The authors crawl the restaurant comment data in China from August 2019 to April 2020 through python.
Step 2: In order to get intuitive and effective analysis results, the authors first cleaned the review data and filtered the stop-words.
Step 3: The authors visualized the review data through word frequency statistics and word cloud graph.
Step 4: Determining the subject words by LDA topic analysis, mined the effective information from the review text.
Step 5: SNOWNLP was used for emotion analysis.

**Figure 1:**
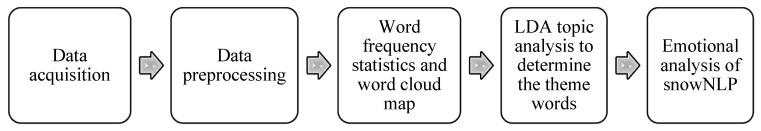
Flow chart of the proposed method.

The flow chart of the proposed method is shown in Figure 1.

## 4. Empirical Research

### 4.1. Data Acquisition

At first, all the cities are split into three groups: high risk area (The cumulative number of patients is more than 10,000), medium risk area (The cumulative number of patients is between1000 and 10,000) and low risk area (The cumulative number of patients is less than 1000) according to the severity of the epidemic. By using stratified random sampling, Hangzhou and Guangzhou were selected as low-risk areas, Beijing and Shanghai were selected as medium-risk areas, and Wuhan was selected as high-risk area. Python programming was used to crawl the catering review data of Dianping platform (https://www.dianping.com, accessed on 21 January 2021) from August 2019 to April 2020. In addition, 20 January 2020 was taken as the cut-off point. Then the data are divided into “pre-epidemic” and “post-epidemic” parts. A total of 11,705 pre-epidemic comments and 2749 post-epidemic comments were obtained (the number of post-epidemic comments affected by COVID-19 was lower than that before COVID-19). The results are shown in Table 1 and Table 2. It should be noted that this paper uses the original language (Chinese) in the process of obtaining, processing, and analyzing the data. We just translated the results into English to show them.

### 4.2. Data Preprocessing

#### 4.2.1. Data Import and Cleaning

It takes restaurant name, various ratings, review content, and review time as result to save them into a file in CSV format. Then it imports the review data into Python and checks whether the data format is correct after import. It preprocesses the data after confirming the success of import. There are many repeated values, modal words, and missing values in the original data, which will lead to deviation between the analysis results and the actual situation. Therefore, it is necessary to delete the repeated values and missing values (referring to the rows without the content), and then take into account the specific situation of the comment data to remove characters such as emoji expressions and low quality review evaluation, etc. Then the text is filtered, and the short sentences below five characters are deleted. Finally, the text is filtered, and the stop-words are deleted by using the stop-words to optimize the text quality and make the analysis results more reliable. Repeat the second data cleaning after the first data cleaning is completed.

#### 4.2.2. Word Segmentation and Stop-Word Filtering

After sentence segmentation processing, the text needs to be filtered. There are a large number of colloquial words which cannot reflect the theme but appear frequently in the comment data. It will affect the experimental results to a certain extent. Therefore, stop-word filtering is needed. In this paper, Jieba [20,21], a third-party natural language processing library of Python, is used for word segmentation of Chinese text, removing stop-words. The result is shown in Table 3.

#### 4.2.3. Words Comparison of Three Risk Groups

At first, the data of low, medium, and high risk areas was analyzed, and this paper got the occurrence of high frequency words, respectively. The specific words are as shown in Table 4 and Table 5:

We can see from Table 4 and Table 5 that the distribution of high-frequency words in three risk areas before and after COVID-19. It is beyond dispute that these words do not have significant differences. What is more, due to the synchronous progress of the prevention and control of the epidemic in different regions after the epidemic, the differences among different regions are small. Thus, this paper combined and summarized the three regions into pre-epidemic and post-epidemic for research and analysis.

### 4.3. Word Frequency Statistics and Word Cloud Map

After text preprocessing, we get the cleaned text data. Then, the word frequency statistics of these processed comments data is carried out. Due to the complexity of Chinese parts of speech, a word may have different parts of speech in different contexts. Therefore, it is necessary to distinguish between word parts of speech in order to ensure their correctness in context. In this paper, POS-Tag is adopted for part of speech tagging of words after word segmentation, and the top ten words with the largest word frequency are selected for easy observation, as shown in Table 6 and Table 7.

Table 6 shows the word frequency statistics and part of speech tagging of comments before the COVID-19, from which it can be seen that the three words most mentioned by consumers are “delicious”, “taste”, and “queue”. The adjective “delicious” ranks first and the noun “taste” ranks second. This indicates that before the outbreak, a major factor for consumers to choose restaurants is the taste of the dishes, and they tend to prefer the restaurants with better dishes. The third is the verb “queue”, indicating that before the outbreak of the epidemic, many popular restaurants need to queue up and wait, which reflects the sound development of the catering industry.

Table 7 shows the word frequency statistics and part of part of speech tagging of comments after the outbreak of the epidemic. It can be seen from it that the three words most frequently mentioned by consumers are “Epidemic”, “less people”, and “food”. Since this article collected data from comments published after January 2020, the outbreak has had a huge impact on residents’ lives, so it is not surprising that the word “epidemic” appears frequently. In second place, it was “fewer people,” indicating the impact of the outbreak on the restaurant industry, with fewer customers in stores. In third place, it was the noun “dish,” indicating that even during the worst of the epidemic, consumers still valued food quality in the restaurant industry. In the review, the word “protection” is a special word for consumers at this particular time. The high frequency of the word “protection” indicates that consumers pay more attention to protection measures when eating out and have a certain panic about the epidemic.

**Figure 2:**
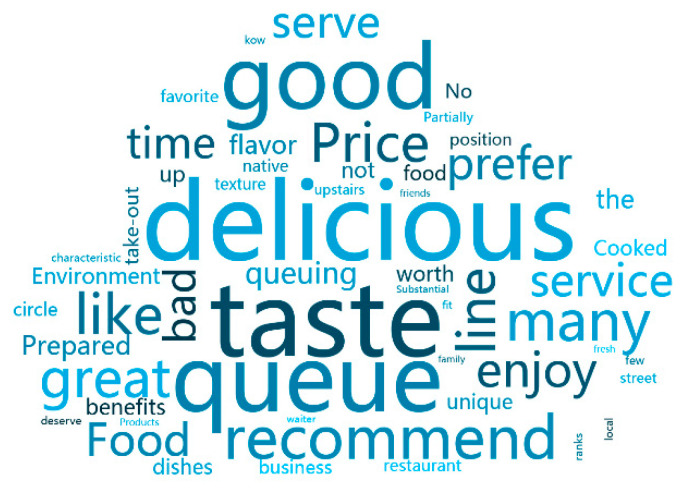
Word cloud before COVID-19.

**Figure 3:**
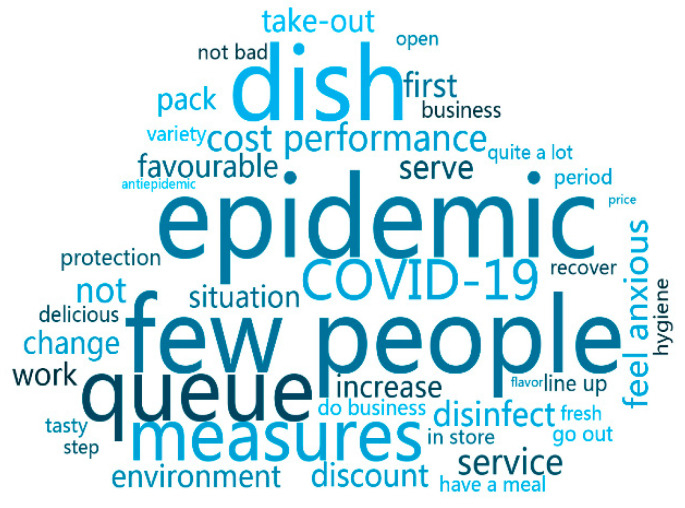
Word cloud after COVID-19.

Based on Python, this paper uses comment word segmentation [22,23] to construct word cloud maps before and after the epidemic (Figure 2 and Figure 3), respectively, so as to make text word segmentation more visible.

The word cloud map makes it more clear that the most common words in these comments, such as “taste”, “service”, “price”, and “feature”, are consumers’ preferences for the restaurant industry before the epidemic; “protection”, “take-out”, and “disinfection” are measures taken by consumers and businesses to reduce the possibility of further spread of the disease during the epidemic. The paper can use word cloud to understand the consumer psychology and consumer behavior in food and beverage during the epidemic period, which is helpful for us to study the psychological behavior of consumers in public health emergencies.

### 4.4. Subject Word Determination

**Figure 4:**
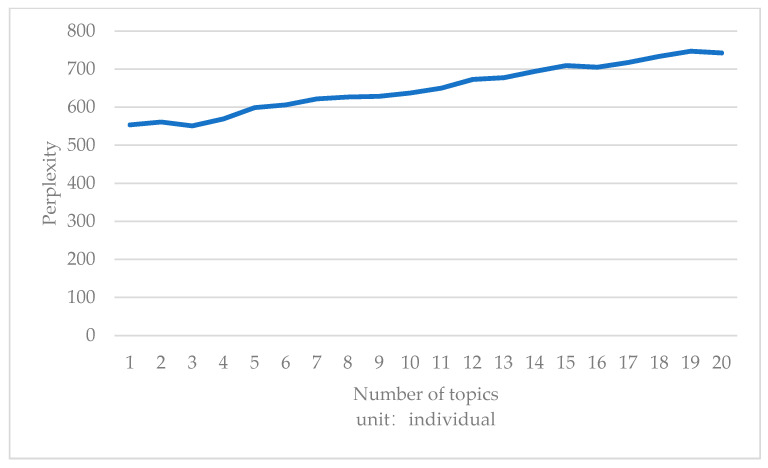
Perplexity of the number of topics before COVID-19.

**Figure 5:**
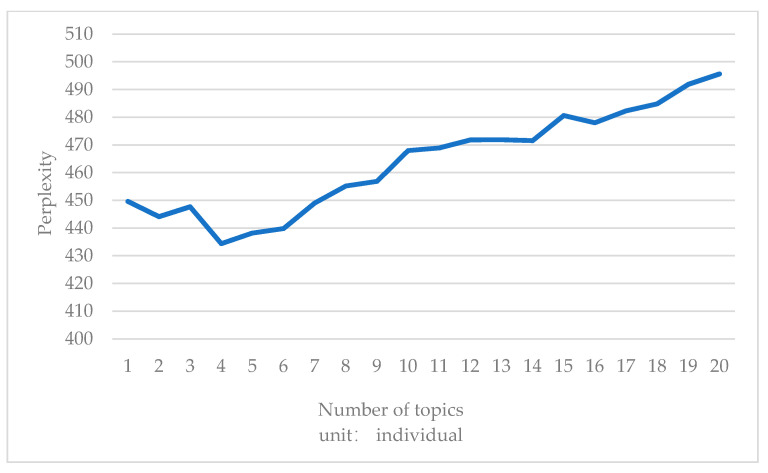
Perplexity of the number of topics after COVID-19.

According to the degree of confusion corresponding to the subject words run by the Python program [24], the following results were obtained: the degree of confusion was the minimum when the number of subjects before the epidemic was three; after the epidemic, the degree of confusion was the smallest when the number of subjects was four. After the epidemic, the reduction of the minimum degree of confusion was greater, so four themes were selected respectively before and after the epidemic shown in Figure 4 and Figure 5.

In this paper, LDA thematic model is used to mine the potential topics of pre-epidemic and post-epidemic commentary texts, respectively. Four themes were selected before and after the epidemic, and six keywords were selected for each theme to get the following Table 6.

As shown in Table 8, subject words were extracted from the comments before the epidemic. In this paper, the first theme is summarized as the quality of dishes in the restaurant. “taste”, “palate”, and “flavor” indicates that consumers pay attention to the taste of dishes, while “fresh” and “meat quality” indicates that consumers pay attention to the quality of dishes. Generally speaking, consumers prefer dishes with good taste and fresh meat quality and pay attention to the quality of dishes. The second theme is summarized as recommended, “clock out”, “for the first time”, “recommend”, and “signs” suggests that many consumers to shop for the first time is usually based on the network or friend’s recommendation.“Web celebrity effect” has a great influence on the choice of consumers, but the probability of words shows that consumers have a high percentage of disappoint web celebrity to web celebrity restaurant and popular dishes. The third theme is summarized as consumer preferences. The words “taste”, “price”, “food”, and “recommendation” respectively represent the demands from different perspectives that consumers value more before the epidemic. The fourth theme is summarized as the restaurant service level, and “speed” and “attitude” grade shows that consumers pay attention to the restaurant’s service, wait speed and the waiter’s service attitude.

As shown in Table 9, subject terms were extracted from the comments after COVID-19. In this paper, the first theme is summarized as the dining style chosen by consumers. Compared with the in-store food, consumers are more likely to choose takeaway, package, or purchase semi-finished products for processing and eating at home. The second theme is summarized dining experience for consumers. Because of the epidemic prevention and control as well as experts for advice citizens out less, most of the food and beverage outlets traffic is small. Moreover, providing eat-in during COVID-19 of food and beverage outlets need reasonable layout according to the requirement, and ensure that diners keep a safe distance, strictly separate portions, etc., so there will be occasional queues. Theme three changes are summed up as food. Because of COVID-19, the transport is limited by certain ingredients. In addition, it is hard to ensure freshness, especially salmon, sashimi, etc.; imported frozen meat, and aquatic products are even more affected. As a result, some food spoilage occurred. Moreover, food and beverage outlets suffered serious losses, and after reopening, many stores chose to reduce the amount of stock, to reduce spending, or to increase their income in order to maintain its normal operation. The fourth theme is summarized as the degree of epidemic prevention. After the outbreak of the epidemic, consumers were required to wear masks, measure their body temperature, use disinfectant to disinfect their hands, and sit at a safe distance when eating. Therefore, there are many comments of this type in the comments.

By comparing the four themes before and after the outbreak, the authors found difference and relationships in consumers’ psychological behavior before and after the outbreak: first, compared with before, after the outbreak of consumers were more focused on the dining environment, paying attention to the epidemic prevention and health conditions of dining place, at the same time also noticing whether the shop, when arranging customers to line up and eat, required them to keep a certain distance. Secondly, the way that the consumers’ diet before, and after, the epidemic has greatly changed. Before the epidemic, they tended to go to online celebrity stores to punch in, but after the epidemic, they tended to pack home or buy takeaway food. Thirdly, before the outbreak, consumers considered whether the store had tasty food, and if the store was a recommend web celebrity restaurant. Moreover, after the outbreak, consumers paid more attention to food safety degree. For example, when some frozen fish tested positive during the COVID-19 outbreak, consumers reduced their demand for such ingredients, so changes to food types also needed to be made accordingly. The relationship between consumer behavior before and after the epidemic was mainly focused on the waiting time for meals (including waiting time and serving speed). All consumers paid attention to the service standard and attitude of the staff in the restaurant. In the highly competitive catering industry, a good service attitude can make a restaurant have more repeat customers and praise rate. Especially during the epidemic, when many stores are closed, good service not only warms customers, but also extends the life of stores.

### 4.5. Sentiment Analysis

**Figure 6:**
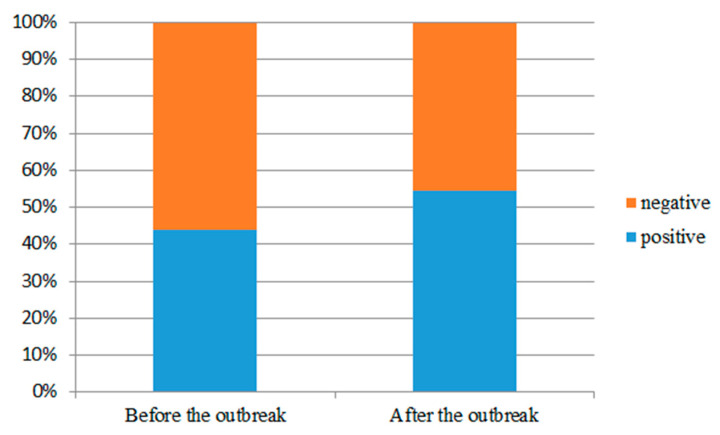
The proportion of emotional tendency before and after COVID-19.

Comments represent the feelings of consumers and can reflect the psychology of their behavior. During the epidemic period, people reduced their consumption outside. Online comments have become an important reference for consumers to choose products, and the emotional tendency of comments is the main factor affecting consumers’ choice. Therefore, after getting the keywords from LDA topic model, this paper uses SNOWNLP [25,26] to judge the situation before and after the epidemic according to the proportion of positive and negative comments before and after the epidemic. The following Figure 6 can be obtained.

Before COVID-19, the positive comments accounted for about 43%, while the negative comments accounted for about 57%. After COVID-19, positive comments accounted for about 54%, while negative comments accounted for about 46%. Compared with before the outbreak of COVID-19, the proportion of positive comments on food and beverage after the outbreak of COVID-19 did not decrease but increased, which is more optimistic, indicating that during the epidemic period, people’s consumption tolerance increased. Their requirements for some basic conditions of food and beverage that they care about before the outbreak decreased. Moreover, they generally had the psychological behavior of expecting the epidemic to end soon and businesses to resume normal business.

**Figure 7:**
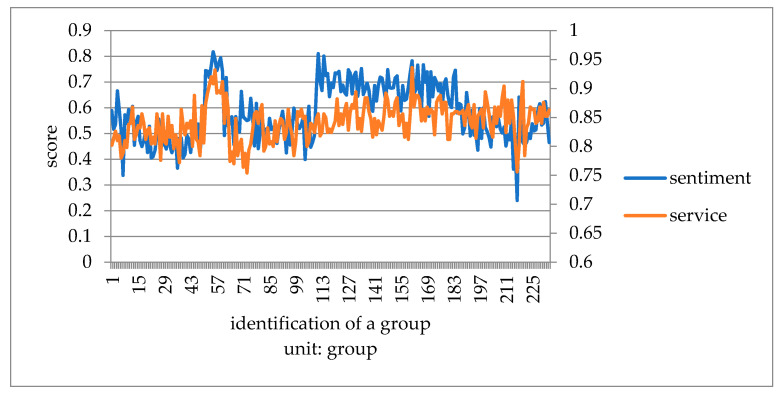
Chart of emotional score and dish taste before COVID-19.

**Figure 8:**
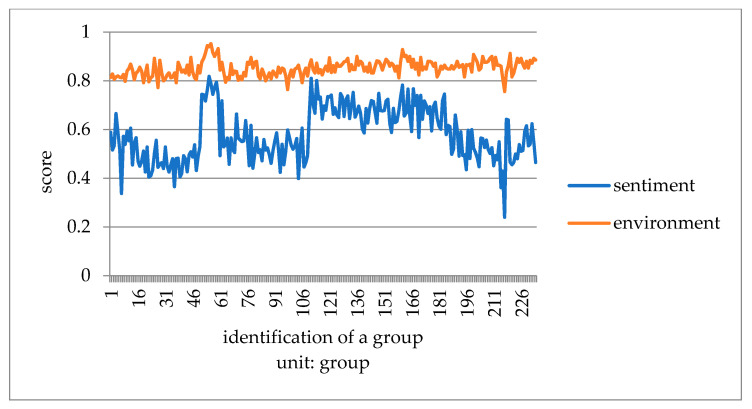
Chart of emotional score and dining environment before COVID-19.

**Figure 9:**
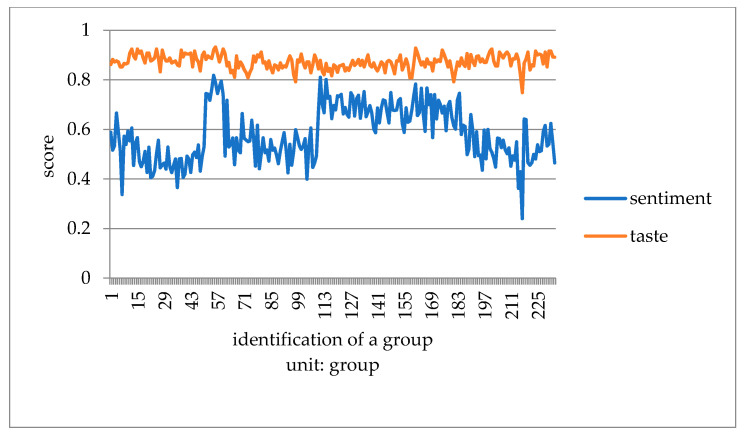
Chart of emotional score and restaurant service before COVID-19.

This paper converts the three kinds of scores of taste, environment, and service obtained by Python into one point system. It compares consumers’ emotional tendency with three kinds of scores to explore which aspect of influence is more similar. It takes the mean value of every 50 data to eliminate the influence of extreme values, and then draws a double line chart to compare with the emotional tendency shown in Figure 7, Figure 8 and Figure 9.

Compared with emotional scores, taste, environment, and service scores are generally higher. The broken line of taste score and emotion score has the lowest fitting degree. Furthermore, it is the most stable among all the scoring lines, indicating that the food taste of restaurants did not change significantly before the epidemic, and consumers’ score of taste fluctuated less. The broken lines of environment and service scores fit well with the broken lines of emotion scores. The peaks and valleys are roughly consistent, indicating that environment and service scores have a greater impact on the overall emotional scores of consumer reviews. Before the outbreak of epidemic, many restaurants did good business. Especially in the dinner rush hour, it is easy to have environmental health problems such as noisy dining environment, overdue cleaning of debris, and foreign bodies in dishes. In addition, there would be long queues, slow delivery of food, and insufficient service personnel leading to lower service standards. Consumer emotion and the emotional score are obviously affected by the environment and service, and therefore arise an obvious peak period.

**Figure 10:**
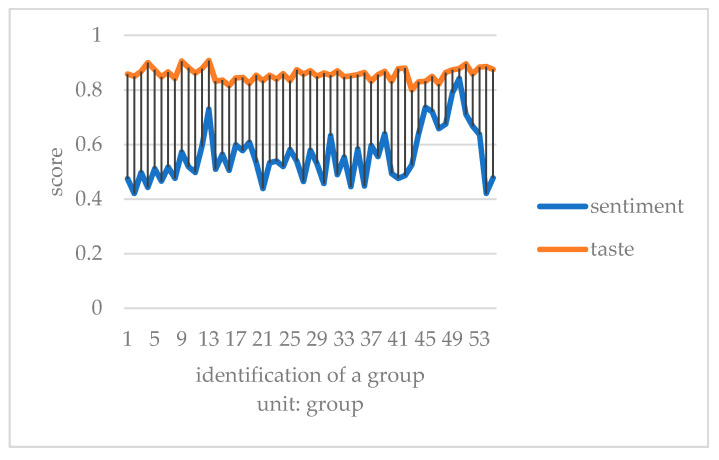
Chart of emotional score and dish taste after COVID-19.

**Figure 11:**
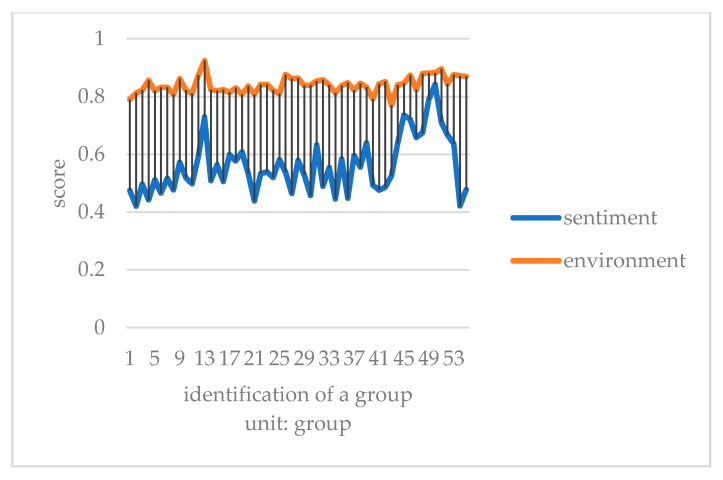
Chart of emotional score and dining environment afterCOVID-19.

**Figure 12:**
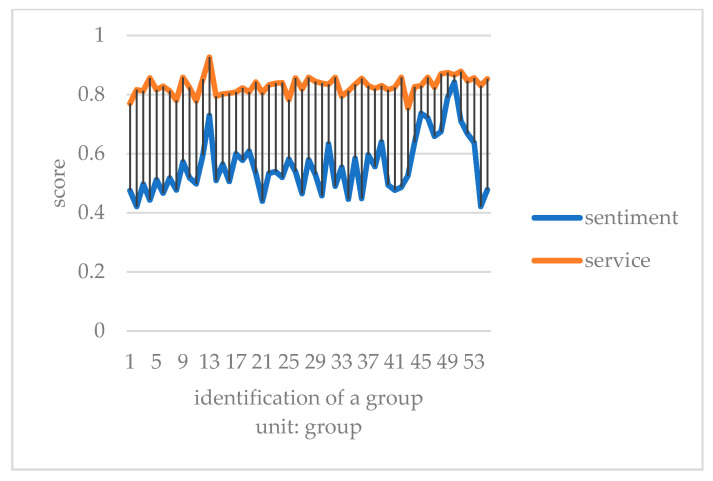
Chart of emotional score and restaurant service afterCOVID-19.

As can be seen from Figure 10, Figure 11 and Figure 12, the scores of taste, environment, and service have a bit gentle trend compared with the scores of emotion, and their mean values are significantly higher than the scores of emotion. Among them, the trend of service score and emotional score are the most similar. Under the condition of stable taste and environment, the service quality of restaurants has a direct impact on the dining experience of consumers. From entering the restaurant, sitting down to leaving the restaurant, the service quality is greatly affected by human factors, such as the quality of the training of the waiter, working hours, and other factors. So it is difficult to maintain a unified standard of service in the restaurant. Whether the waiter can deal with some unexpected situations such as wrong dishes or foreign bodies in dishes, promptly, and solve them smoothly directly affects the consumer’s consumption feelings. Due to the serious loss of catering stores, many stores choose to reduce their stock in order to reduce the cost of storing food or to increase their food prices to increase income to maintain their normal operation after reopening. If the service is not improved in time, consumers are likely to be dissatisfied. Affected by the epidemic prevention and control, the catering stores providing hall food during the epidemic period need to be reasonably arranged according to the requirements to ensure that the diners keep a safe distance. As a result, the number of available seats in the store is reduced, and consumers have to wait at the door of store. Therefore, the waiters not only need to ensure the demands of the consumers who dine in the restaurants are met, but also should pay more attention to the demands of the consumers who line up outside the restaurants.

For example, some measurement including providing hot drinks and seat and so on can be used. Due to the outbreak of COVID-19, the transportation of food materials has been limited to some extent, especially the imported frozen meat products such as salmon and aquatic products, which have been greatly affected. The waiter should inform the consumers of the adjustment of the dishes in the store in time, so as to avoid the change of food materials that may lead to the dissatisfying taste of the dishes, which may affect the consumption experience. In addition, epidemic prevention measures are extremely crucial as well [27,28]. Waiters need to wear masks throughout the whole process, measure the temperature of consumers entering the store and use disinfectant to disinfect their hands to ensure the hygiene of the restaurant. In general, the restaurant needs to ensure the quality of dishes and the environment of the restaurant, increase the management and training of the waiter, and further improve the service level and service awareness of the waiter, so as to bring better dining experience to consumers.

## 5. Conclusions

This paper uses data mining technology to study consumer preferences before and after the epidemic. First of all, through cleaning and sorting of the collected data, the authors get relatively clean comment data. Based on this, the paper carries out word frequency statistics and word cloud mapping and analyzes both psychological behavior and psychological changes of consumers under public health emergencies. After that, this paper conducts a thematic study on comment data [29] and compares the theme composition before and after the epidemic, respectively. It is found that before the outbreak of COVID-19, consumers pay more attention to the taste of dishes when dining out. After this, they focus on epidemic prevention measures, health services, and food safety of stores. BeforeCOVID-19 happened, consumers tended to punch in online stores. However, they prefer pack or order takeout after the outbreak. It seems beyond dispute that COVID-19 not only changed consumers’ lifestyle in many ways, but also changed consumer attitudes and consumption habits. Finally, this paper makes an emotional analysis [30] of the comments by comparing the scores of taste, environment, and service which obtained by Python crawling with those obtained by machine learning. The authors find the relationship between consumer ratings and consumer emotions. During the outbreak response, psychological attitude of catering consumers was still positive. The environment and service had a significant impact on the emotional score of consumers before and after the outbreak, indicating that consumers now think highly of to the state and comfortable degree of dining. Based on the above conclusions, suggestions are as follows:

(1) Improve the level of epidemic prevention services and the transparency of food information to gain consumers’ trust.

Under public health emergencies, consumers pay increasingly attention to the epidemic prevention of restaurants. The catering industry should strengthen the epidemic prevention measures, disinfect on time every day, prepare disinfectant and masks, as well concentrate on cleaning and disinfecting of tableware. At the same time, reasonable arrangements for consumers queuing and dining distance need attention, either. Catering service personnel need regular training to ensure that they can also have professional service standards and warm service attitude in the face of public health emergencies. For example, when consumers are dissatisfied, they can take appropriate measures to appease and compensate, so as to minimize the loss.

From the semi-finished products made from the purchased raw materials to the final products, information such as the name of the ingredients, the place of origin, the place of passage, and the results of various food safety tests are presented on the QR (Quick Response) code. By setting the QR code on the dining table and the network platform, consumers can directly scan the code to understand the changes of the ingredients and the safety of the ingredients. In addition, it can also reduce the time for the waiter to explain the dishes to consumers. Thus, they are available to focus on the service to improve service efficiency while give consumers a sense of security in the event of public security emergencies.

(2) The combination of online and offline double line discount and consumption coupon to win the “heart” of consumer.

The competition in the catering industry is fierce, and all kinds of preferential activities emerge in endlessly. How to stand out in the competition is worth thinking about. Each store can adopt different online and offline discount methods. It can adopt the system of issuing food consumption coupons and exchanging a certain amount of accumulated consumption for dishes online, so that consumers can not only enjoy the discount but continue to consume in the store. At the same time, online sales are more suitable to launch “small dishes” package discount and encourage consumers to buy dishes in the form of package, which can not only save food and beverage stores. The types and costs of food materials procurement can also save consumers’ choice time. Offline preferential activities are more diversified than online ones. Consumers can not only “buy back” by presenting e-coupons to shop customers, but also present their own special dishes or new dishes or store related small gifts to consumers in the form of lucky draw, so that consumers will have a good time; meanwhile, their heart will dance with happiness.

(3) Actively obtain feedback to awaken consumer demand and seize the “stomach” of consumers.

During the epidemic, consumers’ choice and demand for dishes changed. The service personnel should take the initiative to ask consumers about their taste and feelings of the dishes and record the problems so as to rectify the dishes and meet the needs of consumers. Let consumers in the restaurant to obtain “stomach” satisfaction, at the same time to obtain emotional and social satisfaction. Moreover the restaurant can flexibly adjust dishes according to market supply and demand, to give consumers satisfaction.

(4) Reasonable use of community group purchase to provide consumers with “convenience”.

In the context of the epidemic, people generally do not want to wait in line, but want to be able to eat quickly. The community group purchase with the community as the unit arises at the historic moment. The restaurant can accordingly change its business form and flexibly adjust the products it sells according to the market demand. Catering stores can launch meals in the form of group purchase, such as semi-finished products and group purchase packages, in order to meet the living needs of surrounding residents and give consumers convenience.

(5) Develop environmentally friendly takeout mode, leaving consumers with “beauty”.

In the event of public health and security, the increase in the number of people ordering takeaway food has led to the problem of excessive use of disposable cutlery. Catering stores can encourage consumers to reduce the use of disposable tableware by offering certain preferential measures to those who bring their own tableware. Catering stores should replace plastic packaging boxes with degradable paper boxes as much as possible, so as to reduce the harm to the environment and achieve long-term sustainable development.

(6) Data help the recovery of the catering industry.

Local governments can set up data centers and local online epidemic prevention workstations based on “counties’ street” to provide corresponding policy information support for local catering enterprises. At the same time, commercial banks can cooperate with catering enterprises to provide consumption discounts to consumers who handle business in the bank, so as to increase the cash flow of the catering industry and promote the recovery of catering enterprises during the epidemic period.

## Figures and Tables

**Table 1:** Pre COVID-19 data.

Bright Estate Grand Restaurant	4.5	3.5	3.5	It is difficult to get the position of the light estate hotel, yesterday we finally have the chance to fill up a meal.	2020-01-1900:10
Bright Estate Grand Restaurant	5	4.5	4.5	More authentic Shanghai native food, the taste is OK, but heavy.	2020-01-1823:20
Bright Estate Grand Restaurant	4	4	2.5	Shanghai’s food and snacks have characteristics.	2020-01-1822:47
There will be no two	5	5	5	The environment is good. Sitting by the window, it’s quiet. The service is good, taking the initiative to add water. Salmon sashimi: fresh, thick and tender. Overall, it’s not bad.	2019-08-1118:59
There will be no two	5	5	5	First thumb up the service, the service attitude of little sister is very nice. Second dishes, roasted eel is really amazing, it is highly recommended, I feel about sushi is very general, but the autumn fairy tale really good, especially the taste of avocado entrance.	2019-08-1118:43
There will be no two	1	4	4	I saw the comments on the Internet and we were interested in Japanese food, so I chose this restaurant. Overall, the environment is very elegant, the service is very thoughtful, the price is a bit expensive.	2019-08-1117:11
Shrimp Yue Style Restaurant	3	5	5	Environment: The seats are relatively small, very close to each other, no way, too popular.	2020-01-1212:38
Shrimp Yue Style Restaurant	5	3.5	3.5	We entering in the afternoon at 4:30, and were said can eat two hours. However, the sevice is too slow. We have to call the waiter to help urge, too lazy to give a star for it.	2020-01-1211:23
Shrimp Yue Style Restaurant	4	4	4	Haven’t been here for a long time, not a lot of nostalgic food, 7 o’clock to the mango glutinous rice is sold out, spring rolls with special sauce delicious.	2020-01-1209:46
Hangzhou Restaurant	4.5	4.5	4.5	Hangzhou restaurant, has been heard that this is the first time to eat.	2019-12-2320:00
Hangzhou Restaurant	5	5	5	Before the meal, there was just a seat, and when I came out, I was already in line. Two of us ordered five dishes, and we were stuffed	2019-12-2318:17
Xia’s Casserole	4	4	4	The taste is good, the price is right, come in advance so didn’t line up, wait to eat out is two rows of benches are full. It’s worth coming back.	2019-11-0913:33
Xia’s Casserole	5	5	5	It’s not that hard to get a seat, and the dishes are impeccable.	2019-11-0913:02
Xia’s Casserole	4	5	4	Waiting in line for a long time, the place is very large, but a pot of lotus root soup is also very satisfied. Waiting in line all the year round, the business is hot.	2019-11-0911:53

**Table 2:** Post COVID-19 data.

Bright Estate Grand Restaurant	4	4	4	On Monday morning around 10 o’clock, lots of people arein lines. The outbreak is not able to with stand delicious food. In fact, I think buying through WeChat ID, group deals and ordering takeout are also quite good, just not so fresh.	2020-03-3015:26
Bright Estate Grand Restaurant	5	5	4.5	When I was a child living in south maoming road, coming here to eat kept very convenient. During the outbreak, cooked food here such as fresh meat moon cakes and duck arms have been orderly lining up to buy.	2020-03-3011:49
Bright Estate Grand Restaurant	5	5	5	Bright village cooked food that is very famous. Recently, we are still in the process of epidemic prevention and control, so the length of queues relatively good. Today I rarely have business near Huaihai Road, by the way with a point home. Our family’s favorites are their fried pork chops, chicken feet and duck in special sauce.	2020-03-3007:16
There will be no two	5	5	5	In general, there will be no two is not bad, but the price is a little bit high. I usually come here after work. My favorite is the birthday pot bar, which is light and not bland, and then the salad, which is also very refreshing.	2020-03-2116:19
There will be no two	5	5	5	Thisis the most delicious and hygienic restaurant. I have been here many times with my friends. I had my birthday here a few years ago!	2020-03-2111:50
There will be no two	4	5	5	The environment here feel more like western restaurant. I tried “as lemon chicken” and “beef Fried udon noodles” here. “lemon chicken” tastes general, a bit fat. “udon noodles” is good.	2020-03-2300:09
Shrimp Yue Style Restaurant	2	3	3	There exsists thick southeast Asian atmosphere, the light insite is a little dark. service: a lot of people, the table between the table is very close, waiters are too busy to take care of everyone. Also inevitably, their attitude a little anxious.	2020-04-2622:28
Shrimp Yue Style Restaurant	4	4	3.5	The most remarkable feature of his home is that there are many people. When I was in Guangzhou, I had to wait in line for several times, and this time was no exception.	2020-04-2620:17
Shrimp Yue Style Restaurant	4.5	3.5	3.5	There usually have a lot of people in line. Because of the epidemic, there are few people. It is rare that the product is good and the portion is very sufficient.	2020-04-2611:08
Hangzhou Restaurant	5	5	5	The location of the environment is good, the business is booming.	2020-02-0515:15
Hangzhou Restaurant	4.5	4.5	1	Finally eat the Hangzhou restaurant Mizong big pork bun! It’s a very cheap two dollars and five apiece. The whole filling is very juicy and the skin is soft.	2020-02-0514:14
Hangzhou Restaurant	4	4	4	I have been here for several times. Every time some friends come to Hangzhou and say they want to eat authentic Hangzhou food, I bring them to this restaurant. In the lakeside location is very good, after eating can also visit the West Lake and IN77.	2020-02-0513:53
Xia’s Casserole	4.5	4.5	3.5	It tastes great. It’s a web celebrity. It’s really good	2020-03-2601:56
Xia’s Casserole	4.5	4.5	4.5	This casserole is in the hottest dining street, but there are few people during epidemic.	2020-03-2518:40

**Table 3:** Filtering result.

Original Sentence	Results
There are always many people queuing up for the shrimp, but in March, there were fewer people, so I ate the shrimp without allele, which was very rare. They’re all good and the portions are good.	many people queuing up fewer people rare portions good
Because of the epidemic, the store is very quiet, there is not much line.	epidemic store quiet line
The first time I ate out of the epidemic, I felt the same as before. The waiters were all wearing masks, there was hand lotion at the door, and I cleaned the tables more diligently after dinner.	The first time epidemic wearing masks hand lotion cleaned the tables more diligently
At the web celebrity restaurant on Wansong Yuan Road, I heard that the queue was so bad that the kitchen was still preparing the dishes after waiting for half an hour. “Xia Yi Pan Xian” is a bit like a stew pot.	Wansong Yuan Roadqueue was so bad waiting for half an houra stew pot
Kwong Ming Estate’s most famous mooncakes with fresh meat were queued for more than 10 min during the epidemic. The taste of crab is very strong and the skin is crispy. Personally, I think traditional mooncakes with fresh meat are better. Bright Estate’s Green Tuan is the first time to eat.	famous mooncakes with fresh meat queued epidemic taste skin traditional mooncakes

**Table 4:** Distribution of words before COVID-19.

Level of Risk	Words	Frequency
Low-risk area	taste	896
	Delicious	889
	good	630
	compare	493
	recommend	419
Medium-risk area	queue	481
	delicious	556
	taste	466
	good	340
	like	212
High-risk area	delicious	521
	taste	505
	queue	334
	recommend	230
	like	155

**Table 5:** Distribution of words after COVID-19.

Level of Risk	Words	Frequency
Low-risk area	taste	611
	delicious	678
	not	385
	service	264
	queue	175
Medium-risk area	queue	404
	epidemic	245
	semi-finished product	183
	take-out	102
	salmon	89
High-risk area	taste	438
	delicious	411
	queue	256
	epidemic	108
	take-out	84

**Table 6:** Word frequency statistics and part of speech tagging of pre COVID-19 comments.

Word	Frequency	Part of Speech	Word	Frequency	Part of Speech
delicious	2318	*a*	like	925	*a*
taste	2204	*n*	service	871	*n*
queue	1749	*v*	price	862	*n*
good	1584	*a*	flavor	694	*n*
recommended	1069	*v*	environment	652	*n*

Note: *n* refers to noun, *v* refers to verb, *a* refers to adjective, and *adv* refers to adverb.

**Table 7:** Word frequency statistics and part of speech tagging of post COVID-19 comments.

Word	Frequency	Part of Speech	Word	Frequency	Part of Speech
epidemic	1377	*n*	take-out	641	*n*
few people	1297	*a*	not good	516	*a*
Dish	1266	*n*	protection	418	*n*
queue	860	*v*	discount	364	*d*
service	829	*n*	first time out to eat	315	*a*

Note: the words “first” and “not” do not stand alone, but together with other words form meaningful phrases such as “first time out to eat”, “not fresh” and “not tasty”.

**Table 8:** Fourpre-COVID-19 topic models based on Latent Dirichlet Allocation (LDA) Algorithm.

**Theme1 (Quality of Dishes)**		**Theme2 (Popular Recommendation)**	
**Subject Term**	**Probability**	**Subject Term**	**Probability**
taste	0.0369	eaten	0.0255
palate	0.0297	disappoint	0.0194
flavor	0.0189	clock out	0.0150
recommended	0.0133	signboards	0.0111
meat quality	0.0081	first	0.0106
fresh	0.0072	recommended	0.0093
**Theme3 (Consumption Preference)**		**Theme4 (Service Level)**	
Subject Term	**Probability**	**Subject Term**	**Probability**
taste	0.0549	waiter	0.0348
recommended	0.0352	serving	0.0326
palate	0.0225	service	0.0303
price	0.0213	speed	0.0144
cheap	0.0120	allele	0.0132
dishes	0.0096	attitude	0.0070

**Table 9:** Four post COVID-19 topic models based on LDA Algorithm.

**Theme1 (Dining Style)**		**Theme2 (Dining Experience)**	
**Subject Terms**	**Probability**	**Subject Terms**	**Probability**
discounts	0.0168	few people	0.0455
takeaway	0.0149	the firsttime	0.0384
semi-finished products	0.0075	queue up	0.0373
gohome	0.0067	delicious	0.0166
package	0.0022	wait	0.0126
dinein	0.0008	service attitude	0.0057
**Theme3 (Changes in Food)**		**Theme4 (The Degree of Epidemic Prevention)**	
Subject Terms	**Probability**	**Subject Terms**	**Probability**
dish quality	0.0308	attitude	0.0072
fresh	0.0098	epidemic prevention	0.0031
share	0.0095	hygiene	0.0031
surroundings	0.0087	mask	0.0021
salmon	0.0029	Taketemperature	0.0020
sashimi	0.0024	distance	0.0015

## Data Availability

The data used to support the findings of this study are available from the corresponding author upon request.
